# Delayed Presentation of Popliteal Artery Injury Following Tibia and Fibula Fractures: A Case Report

**DOI:** 10.7759/cureus.113511

**Published:** 2026-07-28

**Authors:** Firas Al Majarfi, Yamamah Al Jubori, Muhanna Al-Shaqsi, Hassan Al Lawati, Said Al Kalbani

**Affiliations:** 1 General Surgery, Medical City of Military and Security Services, Muscat, OMN; 2 Orthopedics and Trauma, Medical City of Military and Security Services, Muscat, OMN; 3 Orthopedic Surgery, Medical City of Military and Security Services, Muscat, OMN

**Keywords:** fibula, fracture, popliteal artery, posterior compartment of the lower limb, vascular injury

## Abstract

Vascular damage linked to tibia and fibula fractures is rare; however, it poses a significant risk to limb viability. Disruption of the popliteal artery commonly happens at the time of injury; however, delayed arterial rupture may occur due to a migrated bone fragment, fixation hardware, or rupture of a pseudoaneurysm. We present the case of a 36-year-old man who developed acute, profuse hemorrhage six weeks after open reduction and internal fixation of a distal tibia fracture. The bleeding was caused by a laceration at the popliteal artery bifurcation resulting from a comminuted proximal fibula fracture fragment. Despite immediate restoration of vascular flow by direct repair of the popliteal artery, he subsequently developed severe posterior compartment necrosis and foot drop.

## Introduction

Tibia and fibula fractures are common orthopedic injuries. They are commonly caused by motor vehicle accidents, falls from height, and sports injuries [1]. These fractures are usually displaced and require surgical fixation [1,2]. Complications following tibia and fibula fractures are common and include postoperative infection, nonunion, malunion, vascular and nerve injuries, and compartment syndrome [3].

Vascular damage associated with tibia and fibula fractures is rare; however, it poses a significant risk to limb viability [4,5]. Disruption of the popliteal artery usually presents at the time of injury. However, delayed arterial rupture is extremely rare and may occur due to migration of bone fragments, fixation hardware, or rupture of a pseudoaneurysm [6]. These injuries can initially be asymptomatic and present later with hemorrhage or signs of ischemia [5]. Overlooked arterial injuries pose risks of hemorrhage, compartment syndrome, and subsequent myoneuroischemia [7].

We present the case of a 36-year-old man who experienced acute, profuse hemorrhage from a previously grafted leg incision with a split-thickness skin graft six weeks after open reduction and internal fixation of a distal tibia fracture, revealing an approximately 1-cm laceration at the popliteal bifurcation caused by a comminuted proximal fibula fracture fragment

## Case presentation

**Figure 1:**
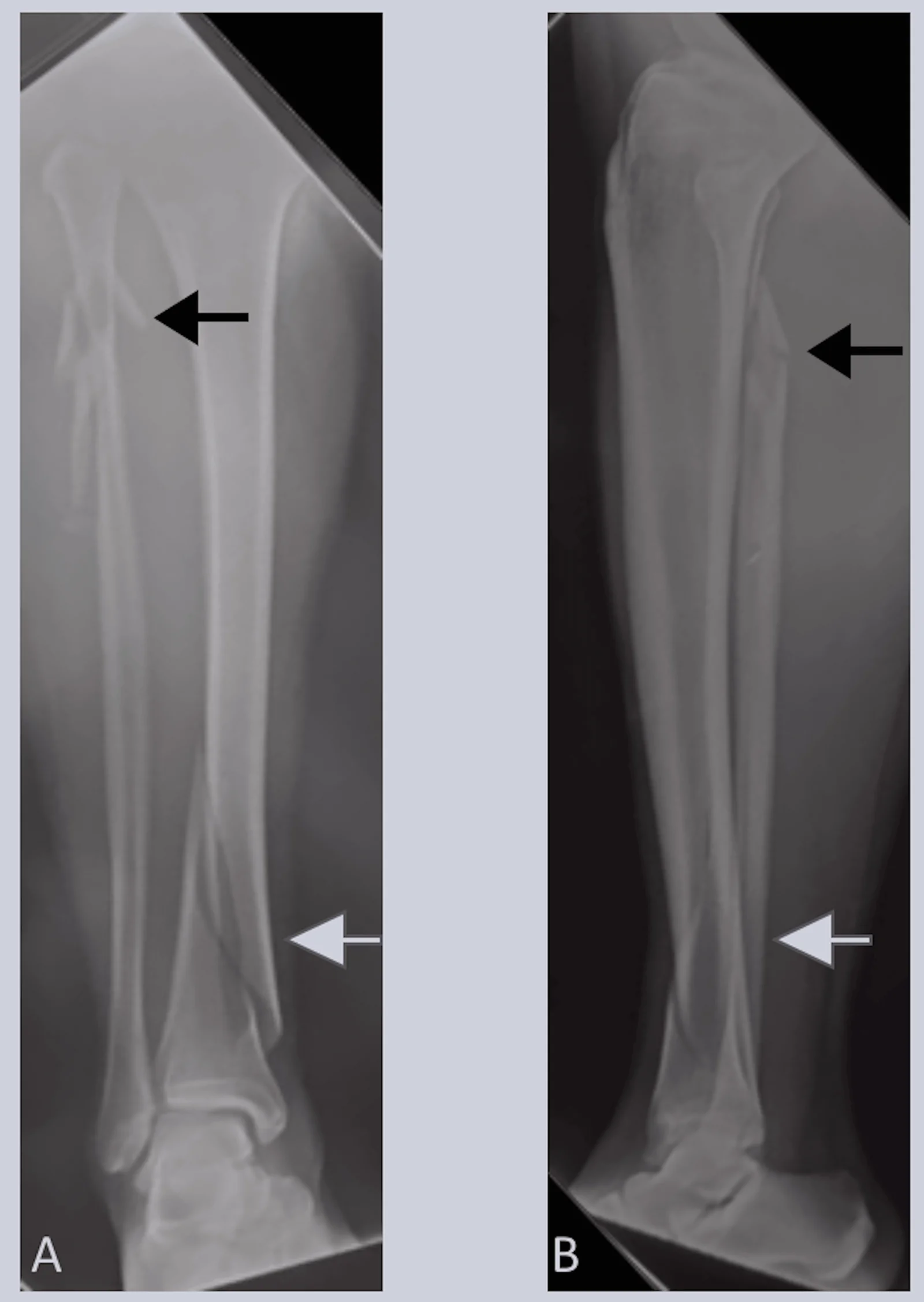
(A) Anteroposterior radiograph of the right tibia and fibula showing a distal tibia spiral fracture (white arrow) and a comminuted proximal fibula fracture (black arrow); (B) lateral radiograph of the right tibia and fibula showing a distal tibia spiral fracture (white arrow) and a comminuted proximal fibula fracture (black arrow).

A 36-year-old man presented to our hospital with right leg pain, swelling, and deformity following a football injury. There was no open wound, and the leg compartments were soft. There were no neurological deficits, and the dorsalis pedis and posterior tibial pulses were palpable. Radiographs showed distal tibia and proximal fibula fractures (Figure 1).

**Figure 2:**
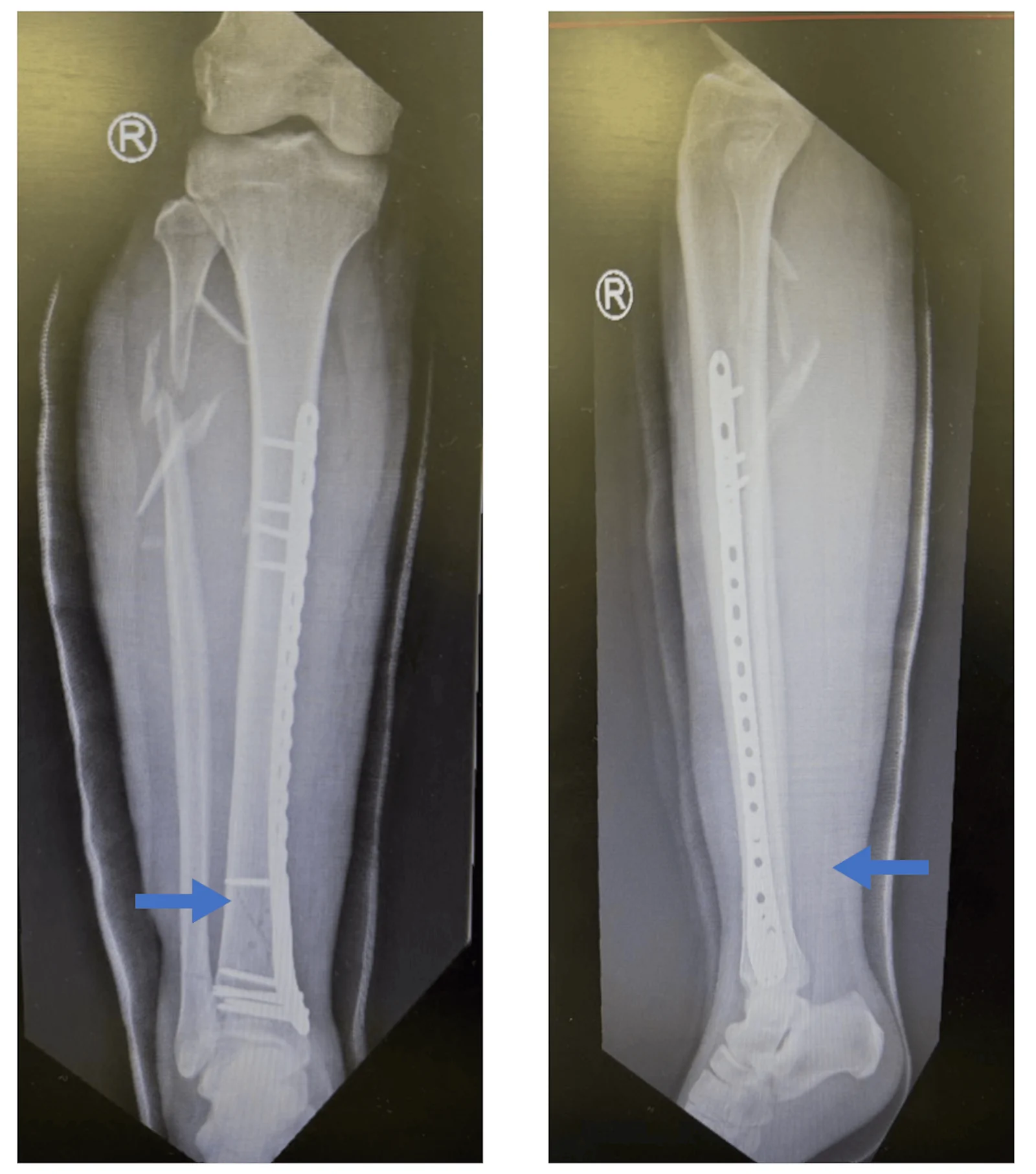
Anteroposterior and lateral postoperative radiographs showing a distal tibia fracture fixed with a locking plate (blue arrow).

The patient underwent open reduction and internal fixation (ORIF) of the tibia fracture using a distal tibia precontoured locking plate, and the surgery was uneventful (Figure 2).

**Figure 3:**
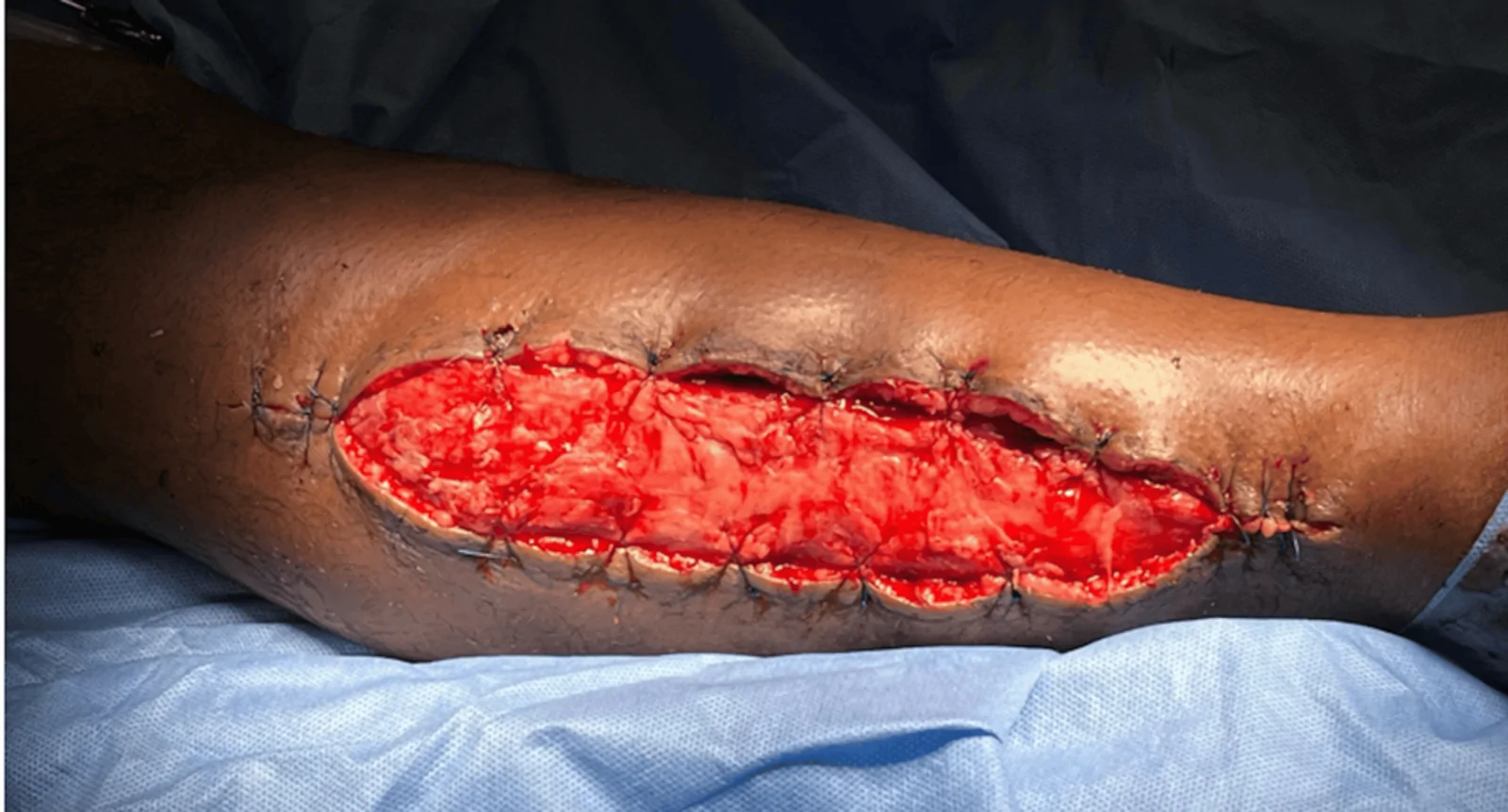
Anterolateral compartment fasciotomy wound.

Unfortunately, he developed compartment syndrome on postoperative day 1 and underwent emergent fasciotomy of all compartments through an anterolateral incision (Figure 3). 

Two weeks after surgery, he developed a fasciotomy wound infection, which necessitated multiple surgical debridements, followed by split-thickness skin grafting (STSG). The patient was discharged home one month after surgery.

Two weeks after discharge, the patient presented to the emergency department with escalating discomfort and edema at the surgical site, accompanied by the sudden onset of profuse bleeding from the grafted wound.

The patient was hemodynamically stable, and examination of the limb revealed a cold, edematous right leg with poorly palpable distal pulses. Handheld Doppler examination identified diminished but detectable signals in the dorsalis pedis and posterior tibial arteries. Computed tomography angiography (CTA) revealed active arterial bleeding at the popliteal artery bifurcation. The posterior tibial artery was patent with normal caliber and contrast opacification. The anterior tibial artery showed attenuation due to extrinsic compression by the surrounding hematoma but remained patent with preserved distal opacification. The common peroneal artery was not visualized at its origin or proximal segment, whereas the distal peroneal artery demonstrated partial contrast opacification.

**Figure 4:**
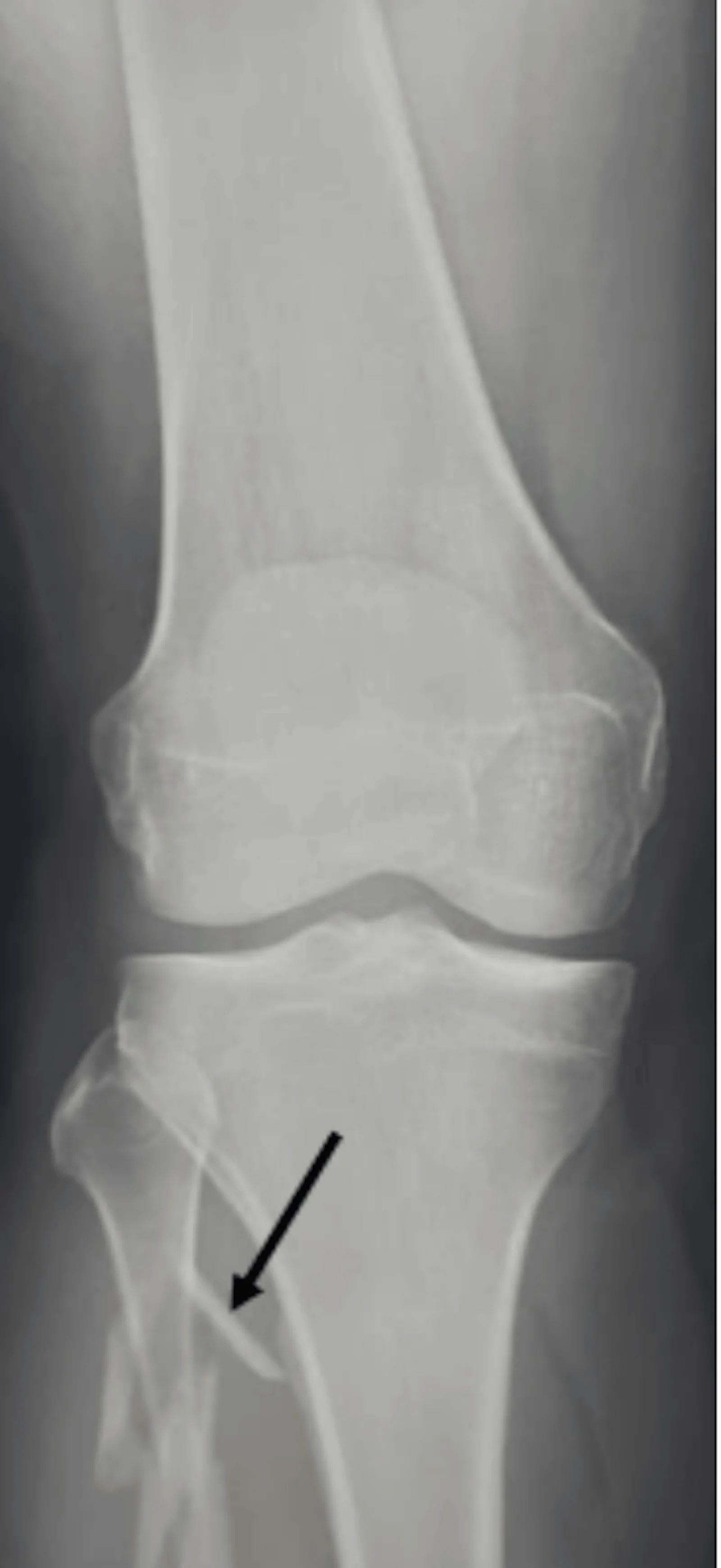
Anteroposterior radiograph of the right knee showing the sharp fragment of the proximal fibula fracture causing injury to the popliteal artery (arrow).

The patient was taken immediately to the operating room for wound exploration. Approximately 1 L of clotted arterial hematoma was evacuated, and a 1-cm tear in the popliteal artery at the bifurcation into the anterior and posterior tibial arteries was identified. A sharp comminuted fragment of the proximal fibula was identified as the cause of the tear (Figure 4). 

The popliteal artery tear was repaired using 6-0 polypropylene. Postoperatively, the patient was found to have foot drop with weak plantar flexion of the toes and ankle. He also developed a deep infection and posterior compartment muscle atrophy and infarction, which necessitated multiple debridements of necrotic tissue, antibiotic therapy, wound care, and hyperbaric oxygen therapy. Subsequently, the wound showed healthy granulation tissue and was free of hemorrhage and purulence. The patient was started on an intensive rehabilitation program.

## Discussion

Arterial injuries after tibia and fibula fractures account for 44% of all bony fractures with vascular injury [8]. These injuries could be a result of arterial spasm, compression by a displaced bone fragment, direct arterial injury, post-fracture reduction, and surgical fixation [8]. Delayed presentation of vascular injury is extremely rare and could be due to injury by a migrated bony fragment, irritation by implants, or rupture of a pseudoaneurysm [9,10]. This case emphasizes the uncommon yet significant event of a delayed arterial rent at the popliteal bifurcation, induced by a displaced fibula fragment. We feel that the sharp bony fragment of the proximal fibula displaced and directly lacerated the popliteal artery. The clinical presentation was severe, characterized by abrupt hemorrhage originating from a previously grafted site. Several fundamental principles arise from this patient's trajectory.

Initially, a heightened index of suspicion for delayed vascular injury is essential in patients with previous fixation who exhibit new swelling, discomfort, or hemorrhage. Despite the presence of palpable distal pulses or maintained Doppler signals, a concealed arterial tear or pseudoaneurysm may exist and rupture unpredictably [11].

The instance highlights the significance of multidisciplinary, damage-control priorities. Swift hemorrhage management, immediate surgical exploration, and conclusive artery reconstruction are critical for limb preservation [11]. Transitory interventions, such as packing, may temporarily mitigate hemorrhage; nevertheless, they are inadequate if the artery wall defect is left unaddressed.

Third, although the restoration of arterial flow is essential, compartmental and microvascular perfusion may still be impaired, increasing the risk of delayed myonecrosis [12]. The deep posterior compartment muscles in this patient underwent infarction days following the first vascular repair.

The significance of supplementary hyperbaric oxygen therapy (HBO₂) is noteworthy in meticulously chosen instances of ischemic or infected wounds with compromised tissue viability. HBO₂ may improve granulation and bacterial management when used alongside thorough debridement, antibiotics, and vascular optimization [13]. The evidence supporting its efficacy is inconsistent and reliant on institutional proficiency; thus, it should be regarded as an addition rather than a replacement for surgery.

Foot drop can occur due to potential complications from hip, lumbosacral spine, and knee surgeries, which are critical to diagnose and manage [14]. The enduring foot drop after this vascular event likely indicates ischemic neuropathy of the peroneal nerve and/or anterior compartment myonecrosis, exacerbated by posterior compartment muscle atrophy, leading to diminished plantar flexion. Initial bracing, physiotherapy, and edema control are the cornerstones of treatment. In the long run, if wounds are stable and infection has been ruled out, tendon transfer treatments, such as posterior tibialis transfer, may be contemplated to restore dorsiflexion and enhance functional gait. Reconstruction decisions must be customized based on muscle viability, remaining nerve function, and individual rehabilitation objectives.

## Conclusions

This report revealed a rare and potentially devastating complication of a proximal fibula fracture. A trauma surgeon should be aware of the delayed presentation of vascular injury in patients with a comminuted proximal fibula fracture. Early recognition is essential, and overlooking these injuries can lead to hemorrhage, compartment syndrome, subsequent myoneuroischemia, and limb loss.
